# Detection of phase‐binning and interpolation artifacts in 4‐dimensional computed tomography imaging using deep learning and rule‐based approaches

**DOI:** 10.1002/mp.70191

**Published:** 2025-12-13

**Authors:** Jorge Cisneros, Nathan H. Feldt, Yevgeniy Vinogradskiy, Richard Castillo, Edward Castillo

**Affiliations:** ^1^ Department of Biomedical Engineering University of Texas at Austin Austin Texas USA; ^2^ Department of Internal Medicine University of Texas Southwestern Medical Center Dallas Texas USA; ^3^ Department of Radiation Oncology Thomas Jefferson University Philadelphia Pennsylvania USA; ^4^ Department of Radiation Oncology Emory University School of Medicine Atlanta Georgia USA

## Abstract

**Background:**

Four‐dimensional computed tomography (4DCT) imaging is a crucial component to lung cancer radiotherapy planning and enables CT‐ventilation‐based functional avoidance planning to mitigate radiation toxicity. However, 4DCT scans are frequently impaired by acquisition artifacts that corrupt downstream analyses that depend on lung segmentation and deformable image registration, such as CT‐ventilation and dose accumulation.

**Purpose:**

This study develops 3D deep learning models to identify phase‐binning artifacts at the voxel level and a heuristic, rule‐based method to identify interpolation slices within 4DCT images.

**Methods:**

We introduce a generator that systematically inserts synthetic phase‐binning and interpolation artifacts into any artifact‐free breathing phase obtained from nine different clinical 4DCT datasets to produce ground‐truth data for (1) training modified nnUNet and SwinUNETR models to detect phase‐binning artifacts, and (2) to determine thresholds in the rule‐based detection method for interpolation artifacts. The use of multiple datasets incorporates robustness across artifact severities, lung geometries, and cancer progressions.

**Results:**

After training on generated synthetic data and several configurations (region‐based learning masks, left–right lung separation), the nnUNet and SwinUNETR models demonstrated state‐of‐the‐art artifact detection accuracy, averaging 0.957 ± 0.024 (95% CI: [0.956, 0.958]), with an nnUNet configuration achieving the highest averaged accuracy of 0.965, sensitivity of 0.805, and specificity of 0.998 when inferring artifact‐affected axial slices from voxel‐level predictions. By interpolating and comparing groups of slices, the accuracy, sensitivity, and specificity of the proposed interpolation detection method is 0.97, 0.97, and 0.97 on manually labeled true artifact cases. We propose a localized artifact correction method that simply replaces the predicted artifact‐affected voxels with the average surrounding lung intensity value, resulting in 65% of lung segmentation masks with Dice scores greater than 0.95 (opposed to 11% of cases before correction) when applying an automatic segmentation tool and within a tight artifact‐bound region. When applied to 1989 cases with true artifacts, SwinUNETR configurations tend to be more generalizable despite marginally lower performance on synthetic artifacts. We quantify this performance without ground‐truth artifact masks by statistically comparing artifact properties of detected synthetic and true cases.

**Conclusions:**

We demonstrate state‐of‐the‐art artifact detection accuracy using 3D deep learning models trained on synthetic data and a rule‐based approach configured on true data, providing interpretability by highlighting which voxel locations indicate a slice is artifact‐affected. The SwinUNETR model accuracy and fast run‐time have the potential to enable more targeted artifact correction methods or signal an imaging technologist when to re‐scan a patient in real‐time.

## 1 INTRODUCTION

Respiratory motion for lung cancer patients creates uncertainty in the location of tumors throughout breathing cycles, hampering delivery of precise radiation therapy.^1^, ^2^, ^3^ Four‐dimensional computed tomography (4DCT) consists of a time‐series of three‐dimensional (3D) CT scans of the breath cycle, providing a “movie” of a patient's pulmonary anatomy while free‐breathing, typically through 10 phases (T00 to T90), with T00 at end‐inspiration and T50 at end‐expiration.^4^, ^5^, ^6^ Since 4DCTs reduce motion‐related uncertainties by accounting for a patient's unique respiratory cycle, they became the standard‐of‐care in the early 2000's^7^, ^8^ and are used routinely for thoracic radiotherapy planning.^9^, ^10^, ^11^, ^12^, ^13^ Although effective, radiation treatment can lead to life‐threatening radiation toxicity.^14^ Functional lung avoidance planning based on CT‐ventilation has recently been demonstrated to substantially reduce the incidence of Grade 2 or higher radiation pneumonitis.^15^ Functional avoidance targets the tumor while minimizing the dose in functional lung, as measured by CT‐ventilation imaging.^16^, ^17^, ^18^ As such, much depends on the fidelity of the CT‐ventilation image, which is known to be sensitive to artifacts.

4DCT acquisition (ciné or helical) involves the continuous and simultaneous acquisition of CT projection data and a breathing signal. CT scanners typically cannot capture the entire thorax region in a single gantry rotation, so data acquisition must span multiple breathing cycles, with images being collected at different couch positions. After scanning, the breathing signal is used to assign respiratory phase information to the projection data, which is then used to reconstruct 3D CT images at the desired breathing phases.^4^, ^19^ Since projection data is sequentially acquired at different couch positions and during different breathing cycles, any irregularities in the patient's breathing patterns can result in artifacts in the reconstructed phase images, hindering the evaluation of lung abnormalities.^20^, ^21^ Phase‐binning artifacts occur frequently, in up to 90% of scans,^22^ with the highest prevalence in near end‐inspiration phases.^23^ Interpolation artifacts, on the other hand, arise in 4DCT imaging when there is an insufficient amount of projection data to accurately reconstruct slices at specific breathing phases and couch positions, often due to irregular patient breathing or from suboptimal acquisition settings. Depending on the scanner and institution, reconstruction algorithms attempt to fill in the missing information by interpolating voxel values from neighboring axial slices, consequently blurring the image.

**FIGURE 1:**
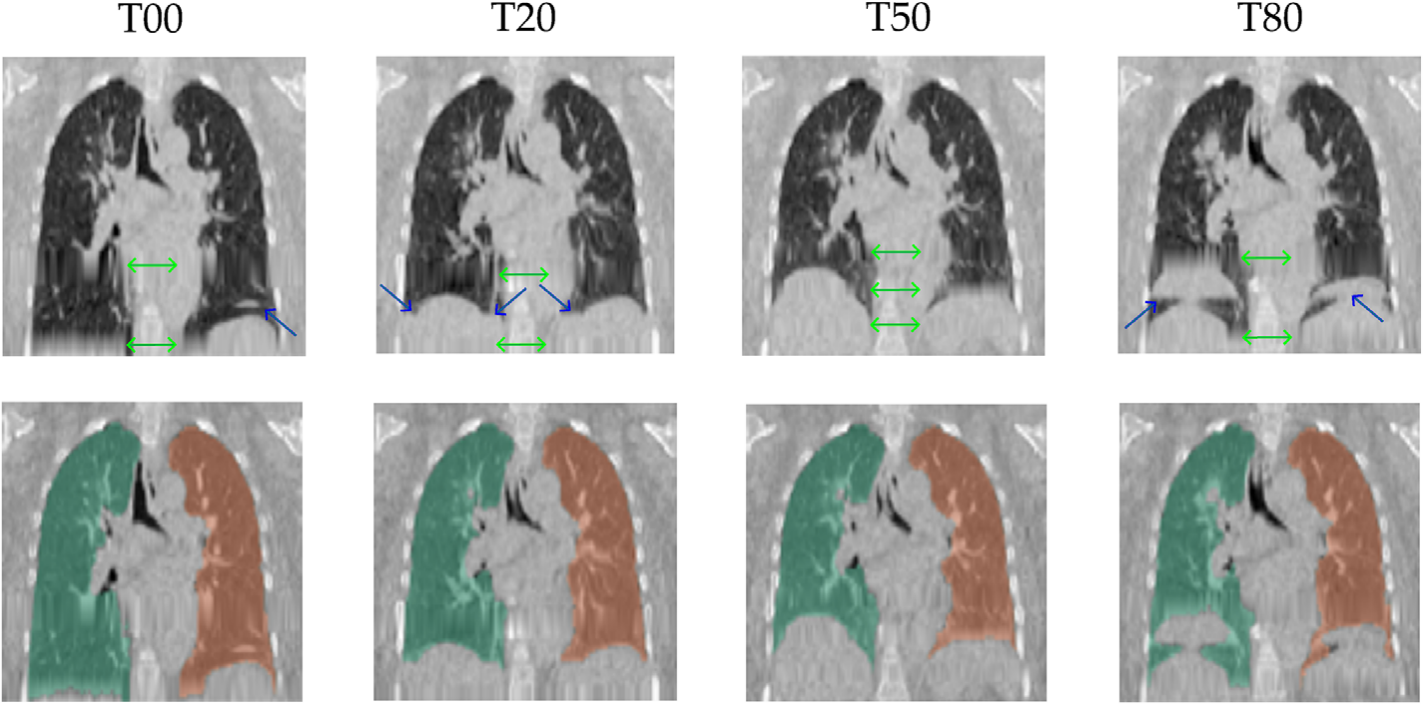
Coronal views of several 4DCT phases (top row) of a patient from an internal dataset, with corresponding left‐right lung segmentation masks (bottom row). Overlapping and duplicated structures (blue arrows) near the diaphragm are clear for T00, T20, and T80 phases, while all phases are affected by interpolation artifacts (green arrows). 4DCT, four‐dimensional computed tomography.

Common artifacts appear as duplicated, overlapping, cut‐off, or smeared structures that degrade the physiological fidelity of downstream analyses, like lung segmentation, patient contouring, and dose calculations.^24^, ^25^, ^26^, ^27^, ^28^ Figure 1 depicts several phases for a patient's 4DCT set, where artifacts are found in each phase.

These impediments highlight the need for effective automated methods for artifact identification. In the last decade, a range of techniques have been introduced for ciné and helical 4DCT phases at the stack and individual slice level.^29^, ^30^ Han et al.,^31^ Castillo et al.,^32^ and Bouilhol et al.^33^ proposed methods using normalized cross correlations to identify artifact stacks by calculating the similarity between adjacent axial slices, followed by a comparison to the similarity of neighboring slices. Slices affected by phase‐binning artifacts show high dissimilarity, while those affected by interpolation artifacts show high similarity, both types appearing as outliers along the axial z‐direction. Although specific implementations vary, this simple method fails to detect artifacts that are still noticeable, like large overlapping structures that blend with the rest of the image.^34^, ^35^ Additionally, it is highly hindered by noisy scans that give false negatives and common cancerous features that give false positives, since tumors typically exhibit an irregular appearance, have variable density, and infiltrate into surrounding tissues. Li et al. improved on previous results using a respiratory motion model to identify artifact stacks in the end‐inspiration phase.^36^

The recent explosion of computational resources and artificial intelligence (AI), specifically deep learning (DL), have led to enhanced artifact localization in CT imaging, but is limited by data availability and computational requirements.^37^, ^38^, ^39^, ^40^, ^41^, ^42^, ^43^ Mori et al. was one of the first to use a DL framework for 2D artifact slice detection in 4DCT coronal slices,^44^ while Shao et al. later developed a 2D U‐Net convolutional neural network (CNN) to detect artifact‐affected regions via bounding boxes from coronal views as well.^45^ Madesta et al. became the state‐of‐the‐art with a 2D fully CNN trained on synthetic data when identifying artifact slices from coronal views.^46^ Most recently, Carrizales et al.^47^ trained a lightweight 2D U‐Net with four encoding layers and four decoding layers to detect artifact‐affected regions in coronal views.

The objective of this study is to develop models for (1) reliably identifying artifact image slices, and (2) identifying the voxel‐level phase‐binning artifacts most likely to affect lung segmentation. To the best of our knowledge, this is the first work to pinpoint phase‐binning artifact‐affected voxels, as opposed to bounding boxes, regions, slices, or stacks, in full volumetric scans. Inference is therefore performed at the building block of an image, but from these voxels, we can infer the larger regions like general artifact‐affected regions or individual slices. This approach paves the way for more accurate artifact correction, improving the utility of a patient's scan for functional avoidance radiotherapy treatment.

In what follows, we present our methodology and results for detecting phase‐binning and interpolation artifacts in any 4DCT phase using AI and rule‐based models, respectively. To achieve high detection accuracy, our models must be robust to lung geometries (including tumors, fibrosis, lobectomies, etc.), CT scanners, and drastic class imbalances between artifact and non‐artifact voxels. However, like many AI applications in biomedical imaging, we are restricted by the availability and diversity of 4DCT datasets, so we develop a generator that systematically inserts synthetic artifacts into an artifact‐free phase given any other phase from the same patient and produces a corresponding 2‐class artifact mask.

By generating diverse training datasets containing various artifacts and their corresponding ground truths, we train 3D neural networks, specifically the nnUNet and SwinUNETR frameworks, to predict phase‐binning artifact masks for a given image. To the best of our knowledge, all previous artifact detection DL models are trained on 2D coronal images. Training on 3D scans leverages inherent spatial and volumetric information, leading to more accurate and robust performance. However, this approach is often infeasible due to large computational requirements and the need for large datasets to mitigate over‐fitting. We train several 3D image configurations of DL models through the use of synthetic datasets. To detect interpolation artifacts, we introduce a heuristic rule‐based approach that analyzes the differences between original and interpolated slices of the same image. The method processes various slice intervals by adding artificial interpolation between the slices and measuring the difference between the interpolated and original image slices. These slice intervals are classified as interpolation artifacts based on criteria and thresholds optimized using both synthetic and manually labeled true (not synthetic) cases.

We also demonstrate the models' performances on detecting artifacts on all phases of clinically acquired 4DCT scans. By having the accurate location of phase‐binning artifact‐affected voxels, we lastly propose a simple artifact correction method that substantially improves the performance of DL‐based automated lung segmentation.

## 2 MATERIALS AND METHODS

### 2.1 Clinical datasets and artifact rank

We rely on a comprehensive collection of nine datasets, critical to ensure models generalize well across different lung geometries and cancer progressions. We use 4 publicly‐available datasets (*4D‐Lung*,^48^ *CT‐PET‐VI*,^49^ *DIR‐Lab*,^50^, ^51^ *POPI* ^52^) that are representative of the other five internal datasets. Table S1 gives an overview of the datasets varying in size, image resolution, image dimensions (before and after pre‐processing), and originating institution. Several patients were imaged multiple times across weeks or months, either before and after surgery (tumor resections, lobectomies, pneumonectomies, etc.) or before and after chemo‐radiotherapy. For those patients with multiple 4DCT scans, we take the first and last to boost the total number of cases, under the assumption that the scans are different enough to prevent over‐fitting during training. All available phases are used to train the DL models for detecting phase‐binning artifacts and to optimize the rule‐based model for detecting interpolation artifacts. Depending on the dataset, all 10 respiratory phases are given or only the T00 and T50 phases. Most datasets are not accompanied by corresponding lung segmentation masks, so for consistency, we compute automatic left and right lung masks for all cases using the recent DL model presented by Nomura et al.^53^ that was shown to outperform previous methods.

With the following hierarchy, we manually quantify images from the clinical datasets according to the presence of artifacts.

- 0 (none): No artifacts are present.
- 1 (mild): Artifacts are present, but do not affect the lung segmentation. Common phase‐binning artifacts are halos, slight overlaps, slight duplicates, or slight incomplete structures. Artifact correction is not necessary.
- 2 (moderate): Artifacts are still subtle, but minimally affect segmentation. There are pronounced overlaps, duplicates, and incomplete structures. Lung anatomy and disease progression are still interpretable, so artifact correction is not necessary in most cases.
- 3 (significant): Artifacts are clear and significantly affect segmentation. Either artifact correction or re‐imaging is necessary.

**FIGURE 2:**
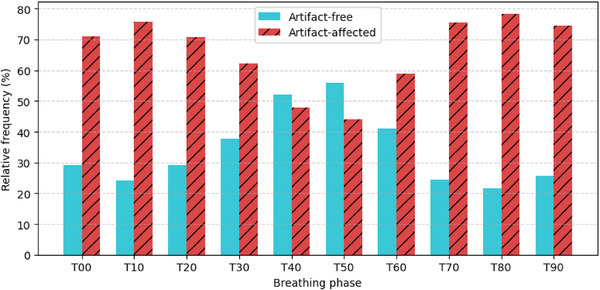
The distribution of artifact‐free and artifact‐affected cases within the nine clinical datasets.

If any interpolation artifact is present, the image is automatically ranked as “moderate” or “significant,” depending on its severity. For practically every dataset, the majority of examined artifacts extensively affect segmentation. In total, the nine datasets yield 427 4DCT cases, where the majority are artifact‐affected. Artifact‐free scans are limited in our compiled datasets, so all phases with ranks “none” or “mild” are deemed “artifact‐free” and used as clean templates for infusing synthetic phase‐binning and interpolation artifacts of severity rankings “moderate” and “significant.” Figure 2 counts how many cases per breathing phase are artifact‐free or artifact‐affected, where the top three phases with the most artifacts, in order, are T80, T10, and T70.

A phase image extracted from any dataset is automatically cropped using a unique bounding box, derived from its corresponding lung mask, that places the lungs in the field of view and removes an abundant amount of background. Hounsfield units are also truncated to [−1024, 600] and then interpolated down to [0,1]. Left and right lung masks are grouped into one mask denoted as $M_{\ell }$ of equal resolution and dimensions as its corresponding pre‐processed image denoted as I.

### 2.2 Synthetic artifact generators and artifact masks

In this study, we introduce two distinct synthetic data generators: one for phase‐binning artifacts and another for interpolation artifacts. These generators are not based on artificial intelligence models, but rather analytical processes that provide significant flexibility in simulating various artifact scenarios.

#### 2.2.1 Phase‐binning artifacts

Given an artifact‐free phase image I, we develop a synthetic data generator that infuses stacks from a secondary phase image $\tilde{I}$ into I to produce image $\hat{I}$ with phase‐binning artifacts in the lower half of the lungs. After selecting an initial uniform stack height h and the number of stacks $N_{\text{PB}}$ to infuse from $\tilde{I}$, a binary slice mask $M_{g}$ is generated that is then smoothed into ${\hat{M}}_{g}$ to allow diffused and blurred artifacts via a Gaussian kernel with standard deviation $\sigma_{g}$, which we refer to as the “artifact visibility” parameter. Artifacts in $\hat{I}$ are more pronounced for $\sigma_{g}<1$ and larger, more faded for $\sigma_{g}>1$. Stacks that were initially specified with height h in $M_{g}$ may have a few more slices for large $\sigma_{g}$ in ${\hat{M}}_{g}$, leading to artifact‐affected stacks of varied heights within $\hat{I}$, representative of artifacts caused by irregular respiration cycles in helical CT acquisition.^31^, ^54^, ^55^ $\tilde{I}$ is also modified into ${\tilde{I}}_{g}$ with a shift intensity parameter $s_{i}$ that shifts axial slices in the coronal and sagittal directions to mimic reconstruction error and noise near the diaphragm. Lastly, we define the weighted difference

(1) $$C={\hat{M}}_{g}⊙\left(I-{\tilde{I}}_{g}\right),$$

where ⊙ denotes element‐wise multiplication. If the condition

(2) $$\sum_{(x,y,z)\;\in \;C}1_{\left\{C(x,y,z)\;\leq \;0\right\}}>\sum_{(x,y,z)\;\in \;C}1_{\left\{C(x,y,z)\;\geq \;0\right\}}$$

holds, then we blend I and the modified ${\tilde{I}}_{g}$ using the feathered mask ${\hat{M}}_{g}$ as

(3) $$\hat{I}=I⊙\left(1-{\hat{M}}_{g}\right)+{\hat{M}}_{g}⊙{\tilde{I}}_{g},$$

resulting in a composite image $\hat{I}$, where $1_{\left\{\cdot \right\}}$ is the indicator function (equal to 1 if the condition is true, 0 otherwise) and 1 is a matrix of all ones. Equation (2) permits I to be any phase image (including T50), generating $\hat{I}$ only if the majority of voxel intensities in C are negative, such that ${\tilde{I}}_{g}$ (and therefore $\tilde{I}$) contains less lung volume (low intensity voxels) or more extrapulmonary tissues (high intensity voxels) than I. This in turn guarantees $\hat{I}$ contains plausible phase‐binning artifacts, like repeated diaphragmatic tissue instead of *missing* diaphragmatic tissue. It also reduces the combinations of I with $\tilde{I}$ to be mostly phases near end‐inspiration with phases near end‐expiration, respectively, while allowing for diverse phase‐binning artifacts across many 4DCT phases. The corresponding lung segmentation mask of $\hat{I}$ is denoted as ${\hat{M}}_{\ell }$.

**FIGURE 3:**
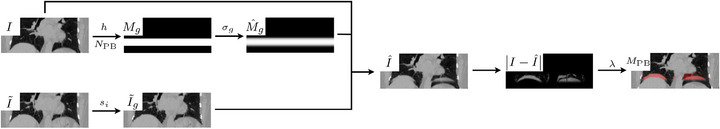
The pipeline for generating phase‐binning artifacts and the corresponding artifact mask. In this example, we select artifact parameters for an artifact‐free T00 phase I, from which a binary slice mask $M_{g}$ is generated that is then smoothed into ${\hat{M}}_{g}$. Next, we take the modified image ${\tilde{I}}_{g}$ of, say, the T50 phase $\tilde{I}$ to infuse I with $N_{\text{PB}}=1$ artifacts, producing $\hat{I}$. Taking $|I-\hat{I}|$ gives general regions of artifacts, but after thresholding with λ, we obtain the artifact mask $M_{\text{PB}}$ (shown in red).

To compute the artifact mask for $\hat{I}$ with phase‐binning artifacts, we first take the absolute difference between I and $\hat{I}$ to isolate voxels that are not present in I. Many of these captured voxels give subtle differences that do not influence segmentation, so by applying a threshold that is linearly proportional to $\sigma_{g}$, we binarize $|I-\hat{I}|$. The lower the threshold, the more voxels of nuanced differences remain. The final artifact mask $M_{\text{PB}}$ is produced after improving artifact delineation by applying a morphological closing operation and connected component analysis.^56^ Figure 3 depicts the full pipeline from phase‐binning artifact synthesis to artifact mask generation.

#### 2.2.2 Interpolation artifacts

**FIGURE 4:**
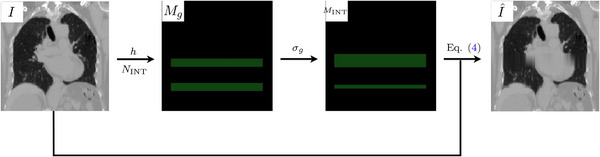
The pipeline for generating interpolation artifacts and the corresponding artifact mask. We select artifact parameters for an artifact‐free T00 phase I, from which a binary slice mask $M_{g}$ of, for example, $N_{\text{INT}}=2$ is generated that is perturbed into the artifact mask $M_{\text{INT}}$ (shown in green) based on $\sigma_{g}$. Equation (4) is then applied to each stack of slices to produce $\hat{I}$.

When projection data is missing, vendors typically approximate the missing axial slices using proprietary methods that extend voxel‐wise Hounsfield units between adjacent, valid slices. Following this principle, we generate $N_{\text{INT}}$ synthetic interpolation artifacts by linearly interpolating axial image slices $I_{z}\left(x,y\right)$ between each pair of selected couch positions $\left[z_{0},z_{1}\right]$ and initial interval length h as

(4) $${\hat{I}}_{z}\left(x,y\right)=\frac{z_{1}-z}{z_{1}-z_{0}}I_{z_{0}}\left(x,y\right)+\frac{z-z_{0}}{z_{1}-z_{0}}I_{z_{1}}\left(x,y\right),\;z\in \left[z_{0},z_{1}\right],$$

resulting in smeared anatomical structures in artifact‐affected image $\hat{I}$, similar to the approach reported by Madesta et al.^46^ and Carrizales et al.^47^ Following a similar strategy when inserting diverse phase‐binning artifacts, we randomly perturb the start and end slice indices with a Gaussian kernel of standard deviation $\sigma_{g}$. This emulates less uniform synthetic interpolation artifacts with more natural variation, where artifact size in $\hat{I}$ are more uniform for $\sigma_{g}<1$ and larger, more diverse for $\sigma_{g}>1$. The corresponding artifact mask $M_{\text{INT}}$ denoting the interpolation artifacts is simply every axial slice within the final couch positions. The placement of these synthetic artifacts is not restricted to specific regions in a scan, like the phase‐binning artifacts. We present the pipeline in Figure 4.

It is important to note that we allow each artifact‐free I to be used multiple times as a template onto which stacks from $\tilde{I}$ are infused and groups of slices are selected for interpolation. We generate different $\hat{I}$ from one I by varying $N_{\text{PB}}$, $N_{\text{INT}}$, h, and $\sigma_{g}$. Deep learning models are tasked to predict the phase‐binning artifacts in $M_{\text{PB}}$, while a heuristic approach is introduced to predict interpolation artifacts in $M_{\text{INT}}$. Each pair of masks are then combined to form the overall artifact mask $M_{a}$.

### 2.3 Detection models

Deep learning is beginning to revolutionize the biomedical fields by enabling automated and highly accurate segmentation of organs and lesions. We build on this momentum by harnessing DL models to segment phase‐binning artifacts. In particular, we capitalize on the recent success of nnUNet and SwinUNETR frameworks, based on their state‐of‐the‐art performance in medical image segmentation tasks and their differing model design choices.

#### 2.3.1 nnUNet framework

The nnU‐Net V2 framework,^57^, ^58^ referred to as “nnUNet,” follows a standard U‐Net structure with deep supervision^59^ and a self‐configuring pipeline that adapts to a diverse range of datasets by optimizing its architecture based on heuristic rules. Each resolution layer consists of two convolutional blocks, each with a convolution, instance normalization, and Leaky ReLU. nnUNet automatically adapts to the dataset by analyzing the training data and selecting suitable patch sizes, allowing it to handle variable input dimensions. It resamples and further crops and pads the input cropped phase image (with unique dimensions, see Table S1) into consistent patches of 128×128×128, making it highly flexible for different data shapes.

When the foreground is significantly smaller than the background, DL models tend to over‐fit on the background using traditional loss functions. To tackle this class imbalance, the nnUNet pipeline over‐samples the foreground, such that at least 33% percent of input images contain a foreground label. During training, nnUNet augments the training data on‐the‐fly to increase the robustness of the dataset with transformations like random rotation, scaling, Gaussian noise, Gaussian blur, brightness alteration, contrast alteration, resolution degradation, gamma correction, and mirroring. The total loss during training is then calculated as the sum of Soft Dice Similarity Coefficient (Soft DSC) loss and Binary Cross Entropy (BCE) loss across all deep supervision outputs. We direct readers to the seminal paper by Insee et al.^57^ for more details, especially on nnUNet's self‐configuring nature.

#### 2.3.2 SwinUNETR framework

The Swin UNETR v2 framework,^60^, ^61^ referred to as “SwinUNETR,” embodies four stages with a convolutional bottleneck feature, where each stage includes a Residual convolutional block, a Swin transformer block, and a final Residual convolutional block.^62^ Each Residual block consists of two convolutional layers, each with a 3D convolution, instance normalization, and Leaky ReLU. At the end of the Residual block, the original input is added to the output. Unlike nnUNet, the transformer's design within SwinUNETR requires input sizes to be multiples of the patch size, so each cropped phase image is resampled before training and inference. To avoid loss of details, each image dimension is resampled to the nearest multiple of 16.

We enhance the SwinUNETR decoder by incorporating deep supervision, adding auxiliary output layers at intermediate stages. After each upsampling and concatenation step, auxiliary convolutional layers produce intermediate segmentation maps that are compared with ground‐truth labels during training. SwinUNETR applies the same data augmentations as nnUNet.

To handle class imbalance, we initially oversampled foreground instances to constitute 80% ($R_{\text{initial}}=0.8$) of each batch and then exponentially decayed this rate starting at epoch 5 ($e_{\text{start}}=5$) toward a target of 40% ($R_{\text{target}}=0.4$) to enhance generalization. This decay followed $R\left(e\right)=R_{\text{target}}+\left(R_{\text{initial}}-R_{\text{target}}\right)e^{-k(e-e_{\text{start}})}$ for epoch $e\geq e_{\text{start}}$, with these specific parameters determined empirically by optimizing validation performance. We found that a high initial foreground oversampling rate allowed for faster initial convergence while decay allowed for greater generalization as the model had the opportunity to see a more diverse set of images. SwinUNETR is also trained using Soft DSC loss and BCE loss across all deep supervision outputs, with normalized deep supervision weights. See Liu et al.^62^ for a deeper discussion and more details on the SwinUNETR architecture.

#### 2.3.3 Rule‐based framework

We present a heuristic, rule‐based approach designed to identify groups of axial slices as interpolation artifacts. Given that detecting these artifacts is a relatively simpler task compared to identifying problematic voxels in phase‐binning artifacts, we propose a non‐AI approach that leverages domain knowledge, providing a transparent and explainable solution without the need for reliance on complex deep learning models.

For any 4DCT phase image, this method identifies potential interpolated slices by artificially simulating the artifact over a slice interval $Z=[z_{j},z_{k}]$ of interval length k−j+1 with k≥j+1. The artificially interpolated interval is then compared to the corresponding interval in the original image. If the original image does not contain an interpolation artifact in that region, the simulated and original slices will differ in intensity and structure. Conversely, if the original image does contain an interpolation artifact, the two intervals will appear highly similar. If the maximum voxel‐wise difference between the simulated and original slices falls below a defined threshold, the interval is flagged as likely interpolated. This operation is repeated over various interval lengths across the entire image to ensure robustness.

In order to guarantee all potential artifact slices are tested, the detection method should iterate through all possible interval lengths that fit within a given image. Although a handful of cases in the nine clinical datasets exhibited single interpolation artifacts as large as 22 slices, there is no theoretical limit on how many axial slices an interpolation artifact can consist of. We initiate the detection method by first testing all slice intervals of the smallest possible interval length (size 3: 1 interpolated slice with 2 interval ends) and then incrementally increase the interval length. Following an exhaustive search and letting the interval length match 2 less the number of axial slices is computationally expensive, especially for large images, and ultimately unnecessary. We note that for large interpolation artifacts, smaller subintervals will consist of highly similar slices and approximate an interpolated artifact, consequently being detected as one. Therefore, if we accurately identify and combine consecutive subintervals of an interpolation artifact, we should eventually recover the full interval of the artifact itself without having to test the full interval itself. After analyzing ground‐truth cases, testing all possible slice intervals of lengths 3 and 4 is sufficient, such that the additional computation time of testing larger lengths outweigh the minuscule improvement in performance. For this reason, we dramatically reduce computation time by implementing a heuristic strategy that only tests interval lengths of size 3 and 4. In the last step, the method filters out redundant or overlapping detections through a simple non‐containment rule and a final merging step.

To further improve performance, thresholds that determine if an interval is likely interpolated or not are assigned to several anatomical regions denoted by dividing the axial dimension into $N_{s}$ equally sized segments. The underlying assumption is that these regions account for local anatomical variability, such as differences in tissue composition and lung volume, which affect the visibility and detectability of interpolation artifacts. This leads to more accurate and rapid detection, since the segments can be run independently.

The thresholds are derived from a data‐driven approach. With ground‐truth slices of interpolation artifacts, we determine the best threshold $p_{i}^{*}$ for each segment $s_{i}$ by solving independent optimization problems that are scalar and bounded:

(5) $$\begin{array}{cc} p_{i}^{*} & =\arg\min_{p_{i}}\frac{1}{N_{Z}^{\left\{i\right\}}}\sum_{j}\;\;D_{L}\left(Z_{j}^{\left\{i\right\}};p_{i}\right),\;\;i=1,\text{…},N_{s},\\ \text{s.t.}\; & 0<p_{i}<1, \end{array}$$

where we minimize the average Dice loss $D_{L}\left(Z;p\right)=1-D\left(Z;p\right)$ across all slice intervals $Z_{j}^{\left\{i\right\}}$ in $s_{i}$ for $j=1,\text{…},N_{Z}^{\left\{i\right\}}$. In this context, the Dice score,

(6) $$D\left(Z;p\right)=\frac{2\cdot \text{TP}(Z;p)}{2\cdot \text{TP}(Z;p)+\text{FP}(Z;p)+\text{FN}(Z;p)}$$

measures the discrepancy between the detected Z and ground‐truth interpolation artifact slices in terms of true positives (TP), false positives (FP), and false negatives (FN) as functions of a threshold p. Based on a few random test cases, we apply tighter bounds $0.2<p_{i}<0.3$ to reduce the search space and speed‐up convergence. This setup allows us to solve the minimization problem in Equation (5) via a brute‐force grid search or a simple optimization solver like the bounded Brent method. Interestingly, because we are applying the same linear interpolation in this method as the one defined in Equation (4) to generate synthetic interpolation artifacts, setting $p_{i}^{*}=\varepsilon \ll 1$ for all segments leads to perfect detection of synthetic datasets, including those generated from the studies by Madesta *et al.* ^46^ and Carrizales *et al.*,^47^ since these works also generate synthetic cases using Equation (4).

The rule‐based framework, combined with a heuristic implementation, makes the detection method inherently parallelizable during both training and inference. This allows for efficient processing of multiple segments across threads, a key advantage when competing with deep learning models that offer rapid inference times. With $N_{s}=10$ segments, the method is comparable with the inference times of the DL models for phase‐binning detection.

#### 2.3.4 Synthetic datasets

Before compiling any synthetic dataset, we first separate clean scans (phases with “none” or “moderate” ranked artifacts) into two groups following an 0.85/0.15 split, respectively: those that will serve as templates to be infused with artifacts and those that are left untouched for testing. The latter group serves as a measure of how well the detection models mitigate false positives. They are not seen during training, because in the presence of input that does not contain labels, gradients can become un‐learnable and deteriorate.^63^ Specifically, when there is a lack of true positives in the artifact mask, loss functions can become highly sensitive early during training and lead to inconsistent and difficult‐to‐learn gradients.

We further impose an 0.85/0.15 split on the template scans to designate the resulting synthetic images for train/test sets. To produce a synthetic dataset, we initially select a range of h, $N_{\text{PB}}$, and $N_{\text{INT}}$ to create a list of possible stack combinations that can be applied to any given template. Then, we cycle through the combinations $N_{c}$ times for each template, every time with different $\sigma_{g}$ values and stack couch positions. We choose $N_{c}^{\left(\text{Tr}\right)}$ for the training set and $N_{c}^{\left(\text{Te}\right)}$ for the testing set, where $N_{c}^{\left(\text{Tr}\right)}\geq N_{c}^{\left(\text{Te}\right)}$, such that the number of cases in the training set is considerably greater than in the testing set to mitigate over‐fitting, while ensuring models generalize to unseen data.

In addition to the paired artifact image $\hat{I}$ and its ground‐truth artifact mask $M_{a}$, we introduce the binary region‐based mask $M_{R}$ derived from the lung mask ${\hat{M}}_{\ell }$ in order for the DL models to focus on the most relevant regions during training and ease the computation burden of 3D segmentation. Moreover, we perform left and right lung cropping to double the overall dataset size. We train nnUNet and SwinUNETR frameworks with or without cropping to $M_{R}$ (denoted as “R”) and with or without left‐right lung cropping (denoted as “LR”). In the Supporting Information, Figure S1 depicts a typical region‐based mask and Figure S2 shows the axial, coronal, and sagittal views of a synthetic image with its corresponding artifact and region‐based masks.

Lastly, we perform k‐fold cross‐validation by further splitting the train set into k folds. The models are then trained and validated k times with a $\left(k-1\right)k^{-1}/k^{-1}$ split from the original training set for each train/validation fold set. Specifically, the DL models are trained on an abundant amount of synthetic data, so we choose k=5. The thresholds of the rule‐based model for interpolation detection are determined from limited true cases with manually labeled interpolation artifacts (see Section 3.1), so we choose k=10.

### 2.4 Performance metrics

Both models are limited to 1000 training epochs, consisting of 250 mini‐batches each, and an early‐stopping condition if there is no decrease in the validation loss after 25 epochs. The networks are optimized using stochastic gradient descent (SGD) with Nesterov momentum (μ=0.99) and an initial learning rate of 0.01 that is adjusted after each epoch using a polynomial learning rate scheduler. SGD is chosen over adaptive optimizers like Adam or AdamW due to superior convergence and model generalization when trained with noisy datasets.^64^, ^65^ All predicted masks are binarized from a probability map by thresholding at 0.85, determined experimentally.

To assess performance on artifacts during and after training, models were evaluated by the Dice score D, accuracy, sensitivity, and specificity. We rely on the following two metrics to identify a high‐performing model: one metric based on sensitivity and specificity, and another on Dice scores. Youden's J statistic^66^ is a standard metric that is bounded between ‐1 and 1. A value of 1 indicates perfect sensitivity and specificity, a value of 0 indicates that the model performs no better than chance, and a negative value indicates a model that performs worse than random guessing. Moreover, the primary goal of training a model on synthetic data is to generalize well to unseen real data, so it is crucial to ensure a model does not over‐fit. Artifacts in the training sets have subtle differences and simplifications that do not capture the complexities of real artifacts, making it difficult for an over‐fitted model to effectively adapt if specific patterns from synthetic data are learned. To quantify the severity of over‐fitting a model, we introduce the index ϕ, defined in terms of the Dice loss $D_{L}=1-D$ for each model's training and testing sets, as

(7) $$\phi =\frac{D_{L}^{\left(\text{Tr}\right)}-D_{L}^{\left(\text{Te}\right)}}{D_{L}^{\left(\text{Tr}\right)}+D_{L}^{\left(\text{Te}\right)}}+1.$$

Bounded between 0 and 2, ϕ<1 implies the model over‐fit to the training set and ϕ>1 implies the model is generalizable. We naturally anticipate the training loss to be lower than the validation loss, because the model has been directly optimized on the training data. A small difference (0.85<ϕ<1) is acceptable, but a large gap (ϕ<0.85) typically suggests over‐fitting. The fold that yields the highest J metric is selected as the best trained fold for a given configuration, while the configuration that performs highest across Dice, J, and ϕ metrics for a given synthetic dataset is selected as the best overall model.

In order to compare our DL models with previous methods, we infer bounding boxes, slices, and stacks from the voxel‐level predictions. To present voxel‐level performance, we report “weighted‐by‐volumes” averages, weighing according to the artifact volume per image to emphasize larger artifacts over smaller ones. We acknowledge the detection performance may be more robust for larger artifact regions, while detecting extremely small, isolated artifact‐affected voxels can be more challenging. However, larger artifacts are clinically more relevant than smaller artifacts, which is why we choose to present weighted‐by‐volume and weighted‐by‐slices averages that emphasize poor performance for large artifacts. For bounding‐box, slice, and stack‐level performances, we report average accuracy, sensitivity, and specificity.

### 2.5 Artifact‐omics

Drawing inspiration from the “‐omics” fields in biology,^67^, ^68^, ^69^ we introduce the term “artifact‐omics” to describe a data‐driven approach for characterizing the size, shape, and orientation of phase‐binning artifacts within a given dataset. For sufficiently large artifacts, artifact‐omics focuses on extracting interpretable features from given artifact masks (either predicted or ground‐truth), like volume, axis‐aligned shape metrics (major, intermediate, minor), elongation ratios, compactness, and sphericity. These features are derived using principal component analysis and region‐based properties in voxel space. Specifically, the major, intermediate, and minor axes are calculated by performing PCA on the 3D coordinates of artifact volumes to find the directions of greatest, middle, and least spread of the data cloud, then converting the variances along those directions into lengths. These lengths describe the size of an ellipsoid that best fits the distribution of the artifact volume. Table 1 displays the full list of features, an overview of how they are computed, and their significance.

**TABLE 1:** Definitions and purposes of artifact‐omics morphological features.

Feature	Definition	Purpose
volume	Number of voxels comprising the artifact region	Quantifies the size of the artifact
bbox_volume	Volume of the 3D bounding box around the artifact	Measures the spatial spread or bounding space of the artifact
solidity	Ratio of artifact volume to its convex hull volume	Indicates surface concavity and irregularity
extent	Ratio of artifact volume to bounding box volume	Assesses how tightly the artifact fills its bounding space
equivalent_diameter	Diameter of a sphere with the same volume as the artifact	Provides a normalized size metric regardless of shape
major_axis_length	Length of the longest principal axis from ellipsoid fitting	Captures the dominant directional extent of the artifact
intermediate_axis_length	Length of the second‐longest principal axis	Describes secondary dimensional spread
minor_axis_length	Length of the shortest principal axis	Reflects thickness or minimal spread
elongation_major_minor	Ratio of major to minor axis lengths	Measures elongation along the primary axis
elongation_major_intermediate	Ratio of major to intermediate axis lengths	Captures deviation from symmetry along secondary axis
elongation_intermediate_minor	Ratio of intermediate to minor axis lengths	Quantifies shape skew or flattening in lesser dimensions
fslatness	Ratio of minor to major axis lengths	Indicates how planar or flattened the artifact is
sphericity	Degree to which shape approximates a sphere	Reflects how compact or round the artifact is
compactness	Ratio capturing shape density, often surface area to volume	Evaluates how tightly packed or dispersed the shape is

*Note*: The Python libraries skimage.measure and sklearn.decomposition were used to extract these features.

By treating artifact properties as measurable phenotypes, artifact‐omics enables statistical comparisons between datasets of artifact features. Specifically, we determine whether the following two groups are statistically equivalent on a feature‐by‐feature basis: (1) ground‐truth and DL‐predicted masks on synthetic test cases, and (2) DL‐predicted masks on synthetic cases and true‐artifact cases. Comparing artifact‐omics of (1) allows us to better understand what types of artifacts the DL models are correctly detecting and potentially which artifacts they struggle with. Comparison (2) serves as a measure for detection performance when no ground‐truth labels are available, like in the nearly 1989 artifact‐affected phases from the clinical datasets presented in this study. Ideally, a handful of experts would identify each artifact‐affected voxel, but this is more subjective and time‐consuming than identifying artifact‐affected slices or stacks. Instead, for a given DL model, we show through artifact‐omics that the predicted artifacts from the *synthetic* test cases are similar to the predicted artifacts from the *true* cases.

In particular, we check for statistical difference between two datasets of artifact‐omics using the non‐parametric Mann–Whitney U test, while checking for statistical equivalence within a specified margin using the TOST (two one‐sided tests) tests. The latter is a statistical method used to determine equivalence between two unpaired groups by evaluating whether the difference between their means falls within a pre‐specified equivalence range.^70^, ^71^ The null hypothesis in TOST posits that the true difference lies *outside* the bounds of practical equivalence. Two separate one‐sided t‐tests are performed: one tests whether the difference is significantly greater than the lower equivalence bound, and the other tests whether it is significantly less than the upper bound. If *both* p‐values are less than the significance level α, the null hypothesis is rejected and equivalence is concluded. Both tests must support that the true difference lies within the equivalence interval. For us, TOST is applied with bounds ±0.2 after z‐score normalization of the artifact properties. We consider two distributions equivalent if both p‐values fall below α=0.05 via TOST *and* if they are not statistically shown to be different via the U test. Because we are testing many hypotheses across different artifact metrics, we apply False Discovery Rate correction to control the expected proportion of false positives and ensure the validity of our statistical inferences.^72^

### 2.6 Localized artifact correction method

The problematic voxels of phase‐binning artifacts manifest as additional tissues embedded into the pulmonary cavity, decreasing the lung volume, and hence the volume of the resulting lung segmentation mask. Many automatic lung segmentation tools fundamentally depend on intensity changes between lung and non‐lung tissues. We propose a localized correction method that simply replaces the intensity values of artifact‐affected voxels in $\hat{I}$ with an averaged intensity value $v_{i}$ of the surrounding lung volume to produce a semi‐corrected image $\bar{I}$. The objective is to improve the artifact‐affected lung mask ${\hat{M}}_{\ell }$ by generating ${\bar{M}}_{\ell }$ that better resembles the artifact‐free $M_{\ell }$. Note that replacing all artifact‐affected voxels with a fixed value does not recover the underlying vasculature and disease progression (if any), such that this method is not intended to fully correct the phase image $\hat{I}$ itself. Instead, it simply camouflages the voxels that cause duplicated, overlapping, and cut‐off structures from a segmentation tool to improve the generated lung masks and additionally serves as a measure of performance for detection.

Phase‐binning artifacts can be interpreted as stacks of axial images that have been incorrectly assigned to the present respiratory phase. This mis‐assignment results in anatomical discontinuities, particularly near the lung base, but typically preserves useful and recognizable features such as the rib cage, main bronchi, chest wall, and spine. On the other hand, interpolation artifacts, wherever they may occur throughout a phase image, tend to blur all anatomical structures that fall between the start and ending artifact slices. Unlike phase‐binning artifacts, every voxel in an interpolation artifact is generally problematic to segmentation, smearing lung boundaries. It is infeasible to predict which voxels within an interpolated stack should adjust their intensity to resemble various unique structures throughout and around the lungs. For this reason, the localized correction method is limited to phase‐binning artifact voxels only. Fortunately, interpolation artifacts are uncommon and mostly confined to helical CT acquisition (see Section 3.1).

To quantify performance of the correction method, we rely on Dice scores D and boundary smoothness β of the resulting lung masks, both evaluated on bounding boxes that enclose detected artifacts. For Dice scores, we compute $\hat{D}$ between artifact‐affected ${\hat{M}}_{\ell }$ and artifact‐free $M_{\ell }$ lung masks to establish a baseline. Then, the Dice score $\bar{D}$ is computed between the corrected ${\bar{M}}_{\ell }$ and artifact‐free $M_{\ell }$ lung masks that measures how effective the correction method is by showing $\bar{D}>\hat{D}$ on average via the Wilcoxon signed‐rank test, a non‐parametric statistical test used to evaluate the differences between two paired samples and determine if their distributions differ significantly.^73^

**FIGURE 5:**
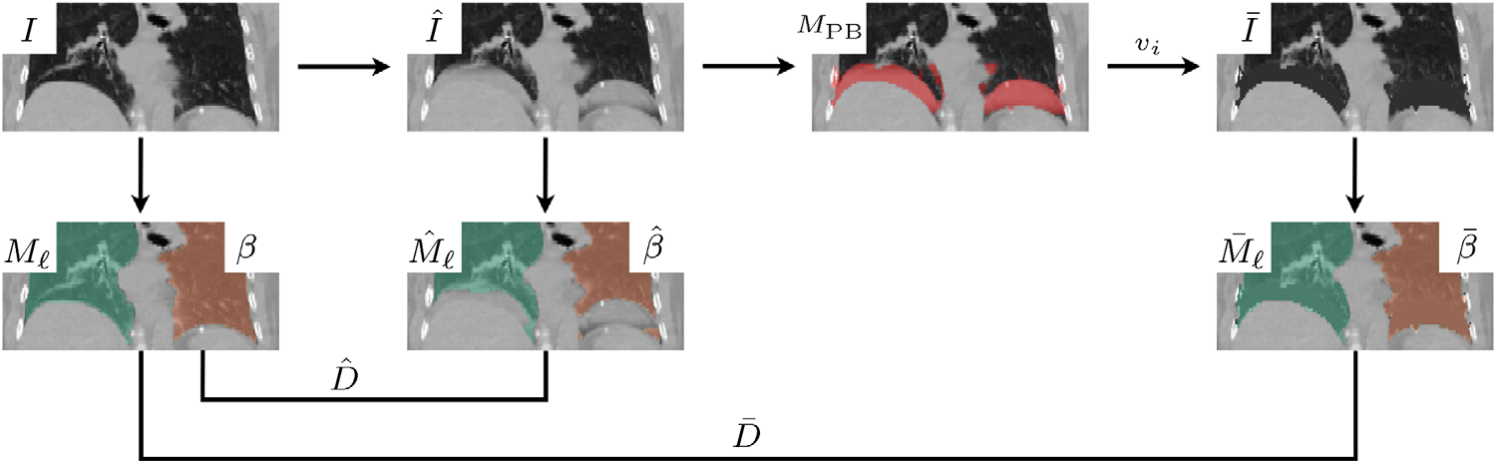
The proposed localized artifact correction method and comparison between Dice scores $\hat{D}$ and $\bar{D}$, and boundary smoothness metrics β, $\hat{\beta }$, and $\bar{\beta }$. Artifacts in $\hat{I}$ drastically corrupt the lung mask ${\hat{M}}_{\ell }$, but significantly improves in ${\bar{M}}_{\ell }$ when replacing the values of detected artifact‐voxels with the average lung intensity value $v_{i}$ in $\bar{I}$.

The boundary smoothness metric

(8) $$\beta \left(M_{\ell }\right)=\frac{S\left(M_{\ell }\right)}{|M_{\ell }|},$$

is simply the ratio of surface area S to volume of a lung's segmentation mask, indicating how jagged the lung mask boundary is, where phase‐binning artifacts in $\hat{I}$ tend to increase a lung mask's surface area and decrease the volume. The 3D surface area of the lung mask within a bounding‐box containing an artifact is calculated from an irregular triangular mesh using Heron's formula for the area of the triangles,

(9) $$S=\sum_{t\in T}\sqrt{s_{t}\left(s_{t}-a_{t}\right)\left(s_{t}-b_{t}\right)\left(s_{t}-c_{t}\right)},\;\;s_{t}=\frac{a_{t}+b_{t}+c_{t}}{2},$$

where T is the set of triangular faces in the mesh and $a_{t}$, $b_{t}$, $c_{t}$ are the side lengths of triangle t, computed as the Euclidean distances between the triangle's vertices. This leads to a boundary smoothness metric $\beta ({\hat{M}}_{\ell })=\hat{\beta }$ that is generally larger than the metric $\beta \left(M_{\ell }\right)=\beta$ of its corresponding clean scan I, and should be similar to $\beta \left({\bar{M}}_{\ell }\right)=\bar{\beta }$ for the corrected image $\bar{I}$. This is verified by the Spearman correlation coefficient ρ, a non‐parametric measure of the strength and direction of a monotonic relationship, linear or nonlinear, between two ranked variables.^74^ Figure 5 presents the localized artifact correction method, along with the corresponding lung segmentation masks, Dice scores, and boundary smoothness metrics. Through an example, we see how double‐structure artifacts degrade the quality of the lung mask and how the semi‐corrected image leads to improved segmentation. The metric $\bar{\beta }$ serves as a measure for both detection and correction performance when no ground‐truth labels are available.

## 3 RESULTS

### 3.1 SYNTHETIC artifacts and datasets

With 1074 total clean images, every synthetic dataset is initialized with 775/138 templates in train/test sets for artifact synthesis and 0/161 templates in train/test sets to be left untouched. Table 2 lists synthetic datasets *S1*–*S3*, all derived using predefined stack heights of h=4,8,16. For h=4, we allowed up to $N_{\text{PB}}=4$ stacks to be inserted into I and up to $N_{\text{INT}}=8$ stacks to be removed and interpolated. For h=8, up to $N_{\text{PB}}=2$ and $N_{\text{INT}}=4$ stacks were permitted, and for h=16, only $N_{\text{PB}}=1$ was allowed, but up to $N_{\text{INT}}=2$ for interpolation. This gives seven possible combinations of h with $N_{\text{PB}}$ to choose from when inserting stacks to generate artifacts. We then cycle through the combinations $N_{c}^{\left(\text{Tr}\right)}=4$ and $N_{c}^{\left(\text{Te}\right)}=1$ times to produce datasets *S1*–*S3*, every time with a new artifact visibility $\sigma_{g}$ and new stack positions to produce one $\hat{I}$ from I.

**TABLE 2:** Synthetic datasets used to train and test artifact detection models, where $\tilde{I}$ denotes the phase from which stacks were taken from.

Synth. dataset	Cases in train/test	$\tilde{I}$ criteria	Avg. volume (%)		Avg. slices	
			PB	INT	PB	INT
*S1*	8174/478	Opposite	2.31 ± 0.05	2.46 ± 0.08	9.3 ± 0.1	9.8 ± 0.1
*S2*	8208/513	Opposite nbhd.	2.12 ± 0.05	2.26 ± 0.07	8.9 ± 0.1	9.5 ± 0.1
*S3*	8468/534	Random	1.71 ± 0.04	2.25 ± 0.07	8.2 ± 0.1	9.5 ± 0.1

*Note*: Means ± 95 CI bounds are shown.

The synthetic datasets only differ by which secondary phase $\tilde{I}$ was used to take stacks from and produce phase‐binning artifacts in $\hat{I}$ following Equation (3) and the condition in Equation (2). We allow I to be any one of the 10 phase images, while $\tilde{I}$, in generating *S1*, was chosen to be the directly opposite phase of I, producing some of the most noticeable artifacts near the end‐inspiration phases out of all three datasets. For *S2*, $\tilde{I}$ was chosen to be a phase in the *neighborhood* of the opposite phase of I, where we define the neighborhood to be the adjacent ± 2 phases of the opposite phase of I. For example, when I is the T30 phase, $\tilde{I}$ is randomly selected to be either T60, T70, T80 (considered direct opposite), T90, or T00. This neighborhood selection criteria allows for more subtle artifacts in $\hat{I}$ than those from *S1*. Lastly, $\tilde{I}$ in *S3* is randomly selected to be any phase other than I. Phase‐binnning artifacts in this dataset are the most diverse, containing some of the most subtle, isolated artifacts than in *S1* or *S2*, evident from Table 2 showing the smallest average artifact volumes and slices, while still containing noticeable artifacts for the DL models to learn from.

**FIGURE 6:**
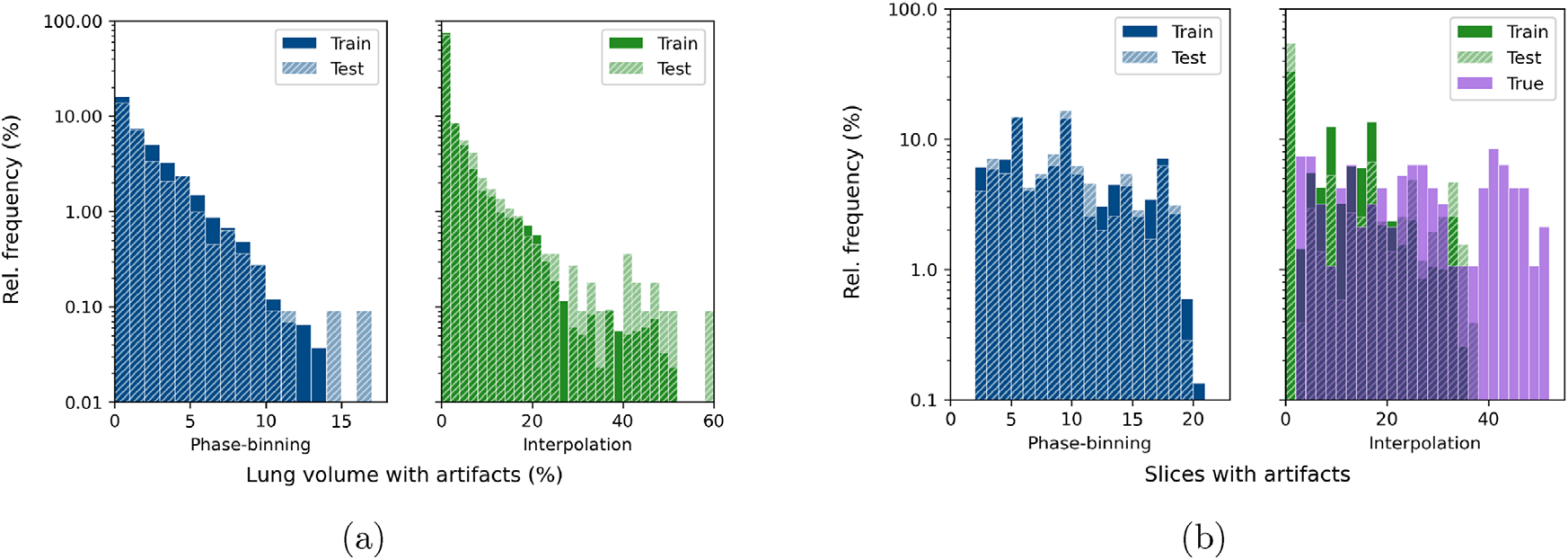
Quantifying artifacts generated in the synthetic training and testing set of *S2*: (a) distribution of lung volumes affected by synthetic artifacts, (b) distribution of artifact‐affected slices, including the 95 cases with true interpolation artifacts from the nine clinical datasets.

Figure 6 quantifies artifacts by computing the artifact‐affected volumes and slices for *S2*, including all of the 95 cases from the clinical datasets with true interpolation artifacts. In particular, there is high concentration of phase‐binning artifact volumes in the range 0.03%–0.2%, steadily trailing off to a few cases with more than 10%. Due to the diversity in scanners and acquisitions from the nine clinical datasets, the thickness of the artifacts were not related to the 4DCT modes. However, with the artifact visibility parameter $\sigma_{g}$, Figure 6 shows the wide diversity in sizes of either type of artifact, from as small as 2 slices to as large as 24 for phase‐binning artifacts and nearly up to 40 interpolated slices in one case. Every synthetic dataset has at least 2 phase‐binning artifact slices, while over 30% of training cases do not have any interpolation artifacts.

### 3.2 DL performance

**FIGURE 7:**
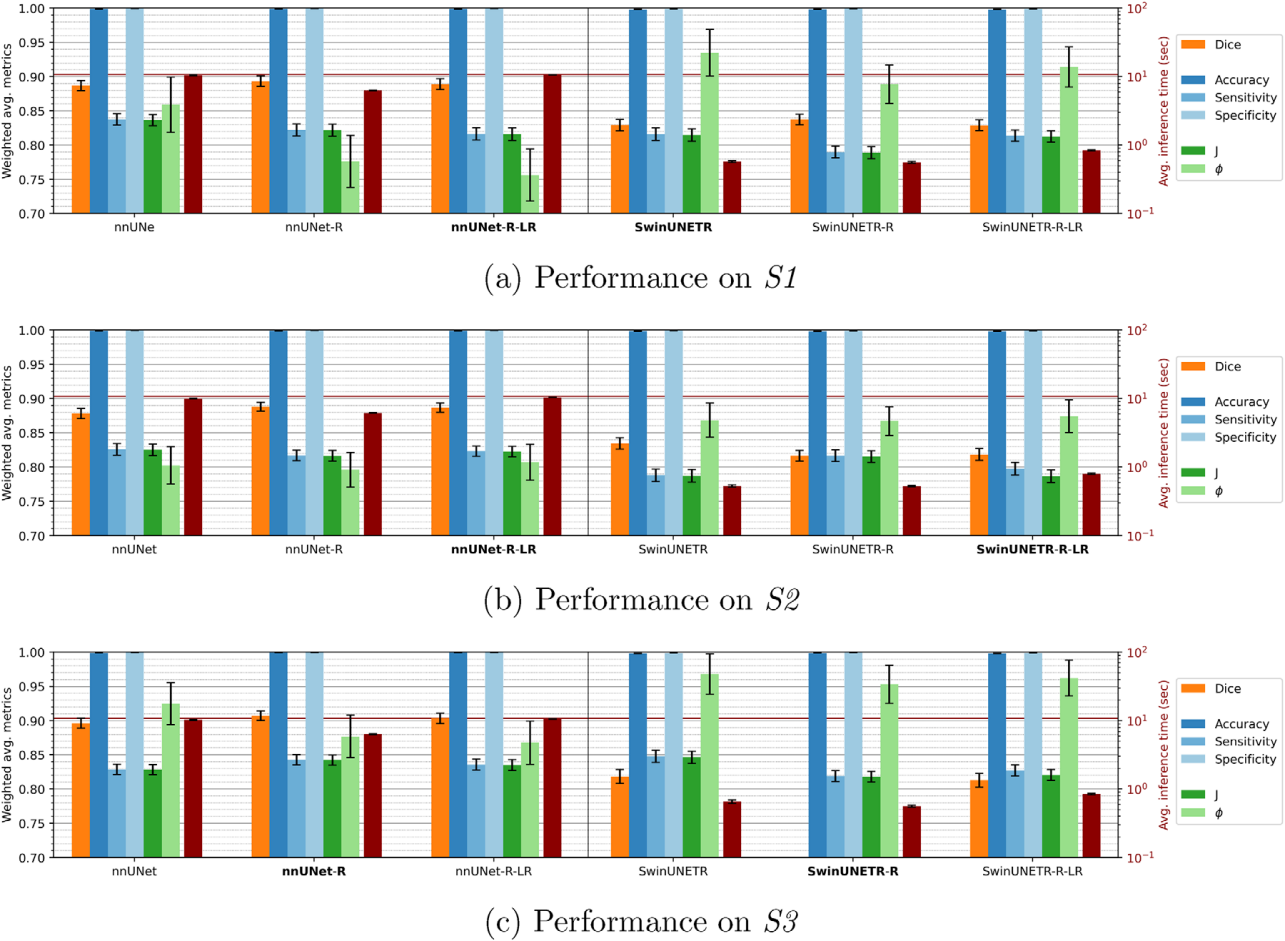
Weighted‐by‐volume average performances of DL models at the voxel‐level on artifact‐affected images across different configurations for each synthetic test set. The segmentation metric is in orange (Dice score), the standard evaluation metrics are in shades of blue (accuracy, sensitivity, specificity), the overall training and testing metrics are in shades of green (Youden's J statistic, over‐fitting parameter ϕ), and the average inference time per scan is in red. The red horizontal line marks 10 seconds. The black vertical line separates nnUNet and SwinUNETR configurations, where the best configurations per dataset are in bold. The error bars denote the 95% confidence intervals.

Supervised learning was performed on an Nvidia DGX machine with 4x A100 80GB GPUs, while inference was subsequently performed on an Nvidia RTX machine with 1x A6000 48GB GPU. Figure 7 displays the weighted‐by‐volume average metrics at the voxel‐level for these model configurations for each synthetic dataset. The model configurations in bold denote the best performing models per dataset. nnUNet configurations slightly outperform those from SwinUNETR, but running inference with the former is more time‐consuming. Configurations with region‐based masks (“R” models) ran the quickest on average because of the localized search space. Configurations with separated left and right lungs additionally (“R‐LR” models) have the most reduced search space, but must be run twice. For example, when identifying artifacts at the voxel‐level, nnUNet‐R‐LR from *S1* gave an average Dice score of 0.87 and sensitivity 0.79 with 10.4 s of runtime per scan, while SwinUNETR‐R‐LR from *S2* gave 0.83 and 0.79 with 0.6 s per scan, respectively. On the topic of time, the rule‐based detection method averages 1.1 s per scan.

We are also interested in how well these DL configurations can identify a clean scan, although none were seen during training, providing further insight into false positive detection. We find that all configurations, except SwinUNETR‐R and SwinUNETR‐R‐LR trained on *S3*, identified a clean scan with false positive rates less than 0.05 at the slice‐level. Table S2 presents these results at the stack, slice, and voxel‐level.

**FIGURE 8:**
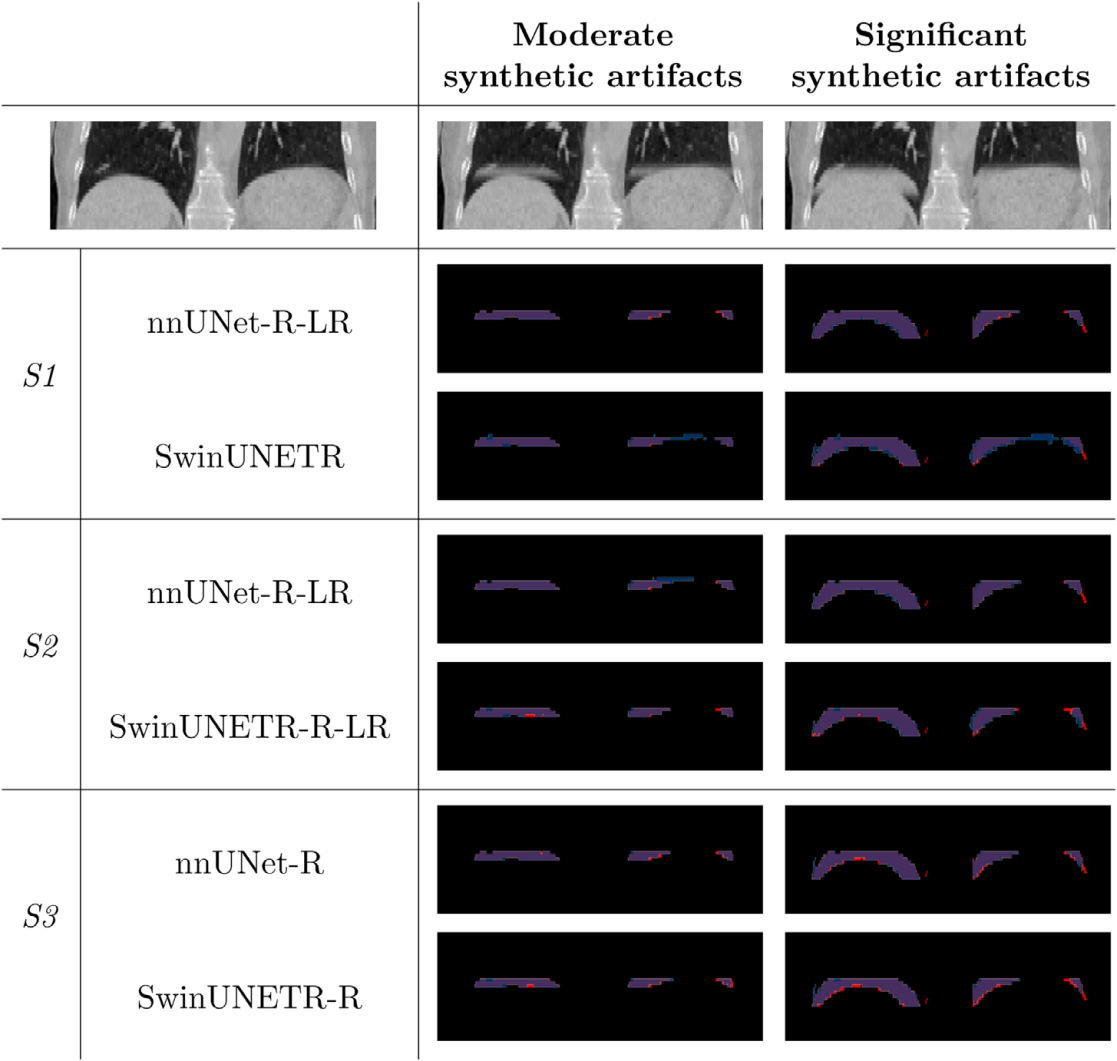
Coronal views of a template and its infused synthetic artifacts with ground‐truth (in red) and predicted (in blue) artifact masks from the top performing nnUNet and SwinUNETR models for each synthetic dataset. Overlap of masks is shown in purple.

Figure 8 illustrates how well each top performing model predicts an artifact mask for a “moderate” and “significant” phase‐binning synthetic case, along with the corresponding clean template that was used for these artifacts. The template was not used to train any of the detection models, such that the lung geometries and infused artifacts were not seen during training. The artifact‐affected cases, however, were selected to carry types of artifacts that every model was exposed to for a fair comparison. Feeding the template into the detection models lead to no false positives, such that the models correctly identified the image as artifact‐free. Moreover, the models perform uniformly across all phases.

All models generally outperform previous studies at the slice and stack level. Table 3 summarizes these results and showcases our performance across averaged accuracy, sensitivity, and specificity. The state‐of‐the‐art detection method was introduced by Madesta et al.^46^ after training fully 2D CNNs to identify synthetic phase‐binning artifact axial slices from coronal slices, performing at a weighted‐by‐slices average accuracy of 0.86, sensitivity of 0.96, and specificity of 0.85 within a bounding box encompassing the lower third of the lungs. For interpolation artifacts, performance was reported at 0.97 across the board. Under similar restrictions, nnUNet‐R‐LR from *S1* once again outperforms all other trained models for synthetic phase‐binning artifacts, reaching an accuracy of 0.99, sensitivity of 0.96, and specificity of 1.00, while SwinUNETR‐R‐LR from *S2* yielded 0.92, 0.81, and 0.97, respectively. On synthetic interpolation artifacts, our rule‐based detection method yields perfect accuracy, sensitivity, and specificity—outperforming any existing interpolation detection method on synthetically generated cases. On cases with true interpolation artifacts, the rule‐based method performed at an averaged 0.97 for accuracy, sensitivity, specificity, and 0.91 for precision.

**TABLE 3:** Comparison of the best performing nnUNet and SwinUNETR detection models with reported average results from recent approaches across artifact stack, slice, and region or bounding‐box detection.

		Stack			Slice			Region		
Study		Acc.	Sens.	Spec.	Acc.	Sens.	Spec.	Acc.	Sens.	Spec.
Han et al. (2011)		—	0.87	0.82	—	—	—	—	—	—
Bouilhol et al. (2014)		—	0.73	0.97	—	—	—	—	—	—
Castillo et al. (2014)		—	0.703	0.476	—	—	—	—	—	—
Li et al. (2017)		0.90	1.00	0.83	—	—	—	—	—	—
Mori et al. (2019)		—	—	—	0.736	—	—	—	—	—
Shao et al. (2021)		—	—	—	—	—	—	—	0.846	0.999
Madesta et al. (2023)		—	—	—	0.86	0.96	0.85	—	—	—
Carrizales et al. (2024)		—	—	—	—	—	—	—	0.78	0.99
*S1*	nnUNet‐R‐LR	0.942	0.875	0.953	0.963	0.789	0.997	0.992	0.745	0.998
	SwinUNETR	0.920	0.882	0.930	0.950	0.805	0.983	0.988	0.748	0.993
*S2*	nnUNet‐R‐LR	0.946	0.882	0.956	0.965	0.805	0.998	0.993	0.765	0.998
	SwinUNETR‐R‐LR	0.934	0.871	0.944	0.956	0.808	0.981	0.988	0.731	0.999
*S3*	nnUNet‐R	0.942	0.849	0.955	0.963	0.766	0.997	0.994	0.700	0.998
	SwinUNETR‐R	0.915	0.873	0.914	0.947	0.794	0.979	0.990	0.695	0.995

*Note*: Not every study detected artifacts at the same level nor reported the same metrics. Madesta et al. only reported weighted‐by‐slices averages.

For the phase‐binning artifact‐omics features, we saw moderately strong Spearman correlations for a handful of variables when comparing ground‐truth with predicted masks for synthetic test cases for all DL models trained across *S1*–*S3*. For example, ρ>0.60 with p<0.0001 for artifact volumes (volume), volumes of the bounding boxes used to enclose the artifact regions (bbox_volume), the diameters of spheres of equal volumes as the artifacts (equivalent_diameter), and secondary directional spreads when analyzing artifacts as ellipsoid shapes (intermediate_axis_length). Specifically for the artifacts predicted by SwinUNETR‐R‐LR trained on *S2*, we present Table 4, where we see the strongest correlations 0.72<ρ<0.86 on these four features. Combining these observations with statistical comparisons via Mann–Whitney U and TOST testing shows that many properties of ground‐truth phase‐binning artifacts vary together with those of predicted phase‐binning artifacts (strong Spearman correlations), but the values themselves differ in scales, offsets, or magnitudes, such that their numerical agreement is not strong enough to declare equivalence (failing Mann–Whitney U testing while passing TOST testing). The four mentioned features with the strongest correlations tend to have varying distributions when using ground‐truth and predicted artifact masks to pinpoint the same phase‐binning artifacts, and therefore exhibiting statistically different behavior (passing Mann–Whitney U testing while failing TOST testing). On the other hand, the family of elongation metrics, extent, and flatness features commonly show Spearman correlations of ρ≈0.5, but are the few that are consistently shown to have equivalent distributions, suggesting they have similar overall behavior yet may not strongly rank individual cases, despite z‐score standardization. Note that some features in the last two columns of Table 4 pass both statistical tests. This occurs when the groups are statistically different (Mann–Whitney U test) but practically equivalent (TOST), meaning the difference is detectable yet small enough to fall within the predefined equivalence bounds. Additional testing with larger datasets may help resolve this apparent inconsistency by increasing statistical power and producing more stable estimates.

**TABLE 4:** Artifact‐omics results when analyzing ground‐truth synthetic artifact masks (“GT synth.”) from the *S2* test set, predicted DL artifact masks from SwinUNETR‐R‐LR trained on *S2* on the same synthetic cases (“DL synth.”), and predicted DL artifact masks on the cases with true artifacts (‘DL true').

				GT synth. vs DL synth.			DL synth. vs DL true	
Feature	GT synth.	DL synth.	DL true	U test	TOST	Spear.	U test	TOST
volume	8543 ± 1378	5156 ± 781	3566 ± 440	Pass	—	0.861	Pass	—
bbox_volume	65497 ± 9001	33537 ± 4584	26044 ± 3306	Pass	—	0.720	Pass	—
olidity	0.263 ± 0.013	0.395 ± 0.015	0.422 ± 0.014	Pass	—	0.467	Pass	—
extent	0.129 ± 0.007	0.169 ± 0.009	0.174 ± 0.008	Pass	—	0.538	Pass	Pass
equivalent_diameter	21.3 ± 1.0	18.0 ± 0.9	16.2 ± 0.6	Pass	—	0.861	Pass	—
major_axis_length	98.1 ± 4.0	75.5 ± 3.4	70.0 ± 2.6	Pass	—	0.583	Pass	—
intermediate_axis_length	49.0 ± 3.1	35.6 ± 2.5	29.2 ± 1.7	Pass	—	0.760	Pass	—
minor_axis_length	6.34 ± 0.36	4.34 ± 0.24	3.98 ± 0.20	Pass	—	0.599	Pass	Pass
elongation_major_minor	18.5 ± 1.0	20.0 ± 1.1	21.3 ± 2.1	—	Pass	0.560	—	Pass
elongation_major_intermediate	2.48 ± 0.13	2.73 ± 0.16	2.95 ± 0.12	—	Pass	0.616	—	Pass
elongation_intermediate_minor	8.21 ± 0.42	8.34 ± 0.44	8.17 ± 0.77	—	Pass	0.477	Pass	Pass
flatness	0.154 ± 0.009	0.148 ± 0.007	0.160 ± 0.006	—	—	0.477	Pass	—
sphericity	7529 ± 741	3605 ± 374	2876 ± 259	Pass	—	0.649	Pass	—
compactness	0.0017 ± 0.0002	0.0036 ± 0.0004	0.0043 ± 0.0004	Pass	—	0.649	Pass	—

*Note*: Means ± 95 CI bounds are shown. All Spearman correlations are statistically significant (p<0.0001).

### 3.3 Localized correction method and true artifacts

Computing Dice scores $\hat{D}$ before correction and $\bar{D}$ after correction affirms that the localized method successfully improves lung masks by camouflaging phase‐binning artifact‐affected voxels. For example, using ground‐truth masks for synthetic cases in *S2*, we find that 16% of artifact‐affected cases have a Dice score greater than 0.95, but substantially increases to 72% after correction. Using predicted masks from SwinUNETR‐R‐LR on the same cases yields similar results with 16% and 65% before and after correction, respectively. In general, there are considerably more corrected lung masks above a 0.95 score than artifact‐affected lung masks. Surprisingly, the simplicity of this approach comes with only a minor loss, where up to 1.9% of lung masks worsened by an average absolute difference of 0.026 when using ground‐truth masks and up to 10% by 0.067 when using predicted masks from top‐performing DL models. Via the Wilcoxon signed‐rank test, we confirm that the distributions of $\hat{D}$ and $\bar{D}$ are different and statistically significant with p<0.0001 for each comparison. The similarity between ground‐truth and predicted masks for each dataset confirms the DL models are performing well and the localized correction method can be extended to cases where ground‐truth artifact masks are unavailable.

**FIGURE 9:**
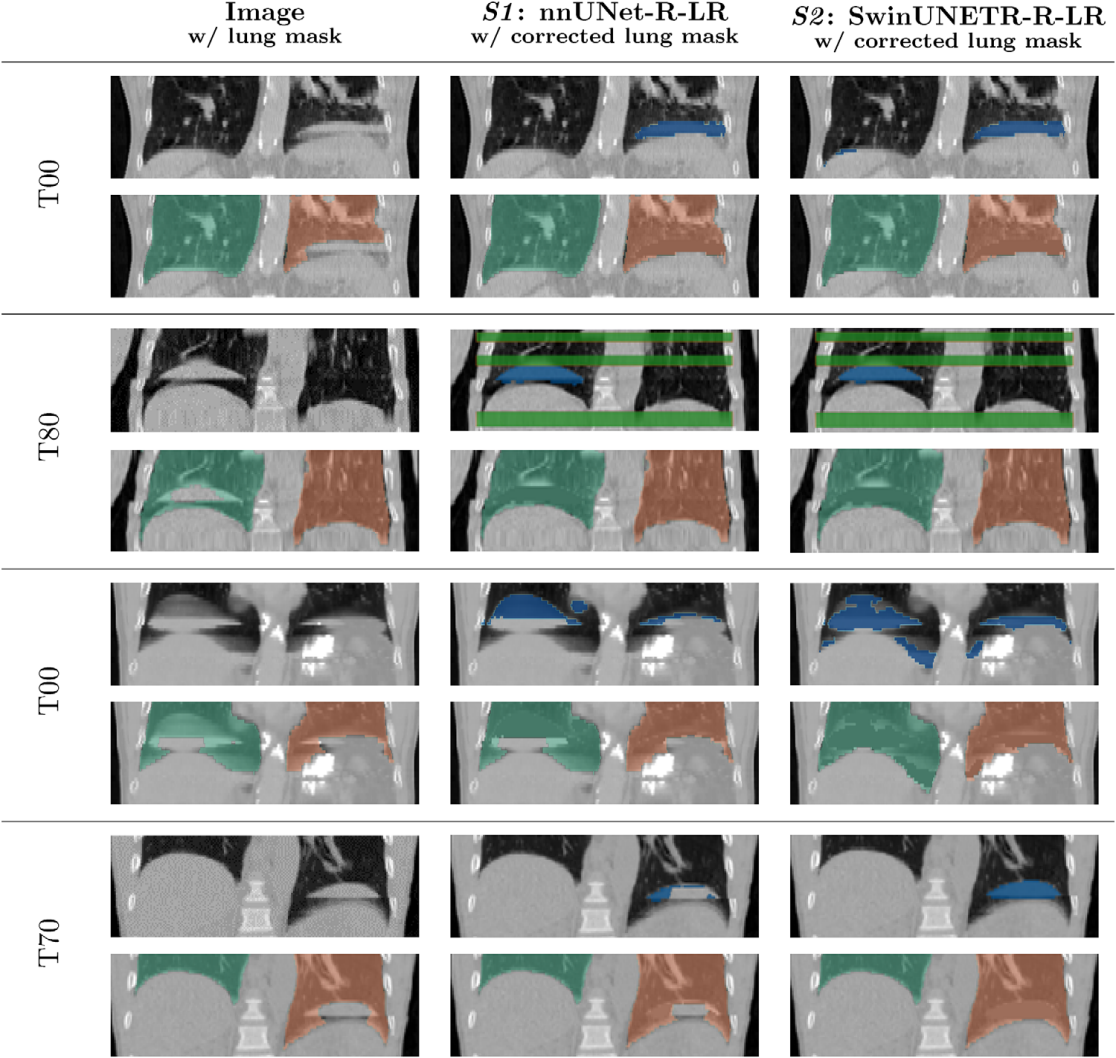
Coronal views of 4 cases with true artifacts and the corresponding lung segmentation mask, along with predicted artifact masks (blue: phase‐binning artifact, green: interpolation artifact) and corrected lung masks.

Figure 9 compares the top nnUNet and SwinUNETR configurations on various phases with true artifacts. Visually, it is easily apparent SwinUNETR‐R‐LR captures larger and more diverse artifacts than nnUNet‐R‐LR, which in turns leads to better lung masks after applying the localized correction method. In the first two cases, both models are able to segment artifacts, even when both phase‐binning and interpolation artifacts are present. However, SwinUNTER‐R‐LR clearly outperforms nnUNet‐R‐LR in the last two cases, even for an artifact of moderate size.

Additionally, we compute the boundary smoothness of artifact‐free, artifact‐affected, and corrected lung masks. Using predicted masks to correct synthetic artifacts leads to similar findings as using the ground‐truth masks. The Spearman correlation coefficient between β and $\bar{\beta }$ is strong when using ground‐truth (ρ>0.92) and predicted (ρ>0.86) artifact masks from either nnUNet‐R‐LR from *S1* or SwinUNETR‐R‐LR from *S2*, ensuring the correction method is highly effective. Applying the correction method to 1989 cases with true artifacts reveals that the distributions of $\bar{\beta }$ values using predicted masks on synthetic and true cases were closest for these two models compared to all others. In fact, the distributions of $\bar{\beta }$ for these 2 models are the only ones that pass TOST equivalence testing. Despite lower performance on synthetic artifacts, SwinUNETR configurations overall tend to be more generalizable to true artifacts than those from nnUNet.

Artifact‐omics provides an avenue to measure how well DL models are performing on cases with true phase‐binning artifacts that have no corresponding ground‐truth artifact masks. If a DL model generalizes well to unseen true artifacts, then the features that are shown to be statistically equivalent between ground‐truth and predicted artifacts of synthetic test cases should also be equivalent between predicted synthetic test cases and predicted true cases, assuming the synthetic data generator outputs artifacts for training that closely resemble the true artifacts. nnUNet‐R‐LR from *S1* and SwinUNETR‐R‐LR from *S2* are the only models that approximately satisfy this condition: all equivalent features from ground‐truth versus predicted synthetic artifacts are not equivalent when analyzing predicted synthetic versus true artifacts, but all equivalent features between predicted synthetic versus true artifacts are equivalent between ground‐truth vs predicted synthetic artifacts. For instance, SwinUNETR‐R‐LR predicts phase‐binning artifacts on *S2* cases with elongation_major_minor (p<0.01), elongation_intermediate_minor (p<0.001), and elongation_major_intermediate (p<0.01) that match the ground‐truth artifacts. The artifact‐omics between the same predicted synthetic artifacts and 1989 true artifact cases shows elongation_major_intermediate (p<0.0001) and elongation_major_minor (p<0.001) are equivalent.

## 4 DISCUSSION

The artifact generator's flexibility to choose stack heights, locations, artifact severity, and from which secondary phase $\tilde{I}$ to take stacks from gives rise to diverse synthetic artifacts that are representative of true artifacts. Although clean templates were repeated $N_{c}^{\left(\text{Tr}\right)}=4$ times when generating synthetic artifacts, Figure 7a–c shows that repeating each clean scan surprisingly does not lead to over‐fitting, generally not learning specific patient lung geometries and disease progressions. Figure 7a,b shows that by diversifying the infused stacks for phase‐binning artifacts leads to an increase in performance, but Figure 7c implies diversifying too much is possible, leading to poor performance. The artifact‐omics comparisons show that *S3* is so diverse in its artifacts that all ground‐truth features are statistically different (p<0.05) from those determined by predicted artifact masks. The lack of consistency in the training data of *S3* prevents DL models from learning which voxels in a phase image $\hat{I}$ are phase‐binning artifacts or not.

Overall, our experience indicates that diversity is beneficial up to a point: modest variation in stack parameters promotes generalization, but excessive variation (as in *S3*) prevents the network from learning consistent artifact patterns. Thus, parameter sensitivity is most pronounced in artifact severity and phase selection, with stack geometry acting as a secondary factor. These findings emphasize the importance of striking a balance between realism and diversity when generating synthetic training data.

Although both nnUNet and SwinUNETR frameworks take into account class imbalance in their respective manners, it appears that nnUNet over‐fits to the training data more than SwinUNETR. While nnUNet's oversampling technique can help mitigate over‐fitting, performance is not as high as desired, because small foregrounds can be difficult to train on with objective functions like Dice and BCE losses. Bouilhol et al. perform non‐AI detection also for an image on separated left and right lungs, with the goal of preventing over‐fitting by limiting sensitivity to anatomical changes and augmenting the training sets.^33^ We found over‐fitting was slightly reduced (higher ϕ metric) in most cases when following this approach for our DL detection models with LR configurations, but performance varied: only models trained on *S2* improved performance. This hints at the notion that although common artifacts, like double‐structures, may not be present on both sets of lungs, the subtle differences caused by a misplaced stack can be detected and aid in identifying artifact‐affected voxels.

Table 3 confirms our DL configurations outperform previous approaches overall. Our DL models surpass the state‐of‐the art accuracy and specificity at the slice level. Specifically, Mori et al.^44^ generated synthetic artifacts only from phases near T00 in the breath cycle, reaching an accuracy of 0.736. Prominent artifacts are commonly caused by misplaced stacks from phases opposite to T00, so it is encouraging to see our DL model configurations yielding a higher averaged accuracy after being trained on synthetic training sets that picked the secondary phase $\tilde{I}$ as directly opposite or from a neighborhood opposite to I. Like previous methods reporting stack‐level metrics, we did not obtain consistent results across accuracy, sensitivity, and specificity. The detection approach with the highest sensitivity (1.00) gave a low specificity of 0.83, while the approach with the highest specificity (0.97) gave a low sensitivity of 0.73. With any of the nnUNet or SwinUNETR frameworks, we acquired accuracy and specificity all greater than 0.91, but sensitivity was frequently the lowest‐performing metric, most evident when inferring bounding boxes with a sensitivity of 0.695. Shao et al. predicted artifacts at the regporting an averaged sensitivity of 0.846 on only three scans (each with sensitivity of 0.908, 0.874, 0.755),^45^ while Carrizales et al. reported sensitivity of 0.78 and precision of 0.58.^47^ Although all of our DL methods achieve an averaged precision of 0.88 or greater at the region‐level, our noticeably poorer performance can be attributed on how the bounding boxes are inferred from detected voxels, where the process of aggregation can introduce errors or inconsistencies. For example, if the DL model detects parts of the same artifact as separate clusters of voxels, they might be aggregated into multiple small bounding boxes instead of a single, larger bounding box. This can result in poor overlap with the ground truth bounding boxes. Nonetheless, we demonstrate exceptional performance at the stack and slice levels that are more relevant for artifact correction methods. The speed and accuracy of the rule‐based method and the SwinUNETR framework suggests the potential to alert imaging technologists in real‐time when a re‐scan is necessary, reducing costs and time for both patients and clinics. By achieving high levels of accuracy, specificity, and generalizability, the study contributes valuable insights into developing data‐driven detection tools for medical physics, potentially improving patient outcomes.

For our proposed localized correction method, results based on Dice scores and boundary smoothness ratios imply: (1) the method is effective and significantly improves lung masks, (2) the method worsens a few cases with predicted masks more than ground‐truth masks, but the average drops in performance are negligible, and (3) the DL models generalize well and can identify unseen synthetic and true artifacts. Specifically, nnUNet configurations perform better than SwinUNETR configurations on detecting synthetic artifacts, but vice‐versa when detecting true artifacts. SwinUNETR‐R‐LR trained on *S2* was deemed the best overall model after its notable performance on true artifacts, even in the presence of tumors or fibrosis. nnUNet and SwinUNETR frameworks were both trained on diverse artifacts with various stages of cancer, but the former noticeably over‐fitted to the synthetic data and failed to generalize as well to true artifacts, perhaps due to its oversampling technique or 30.5 million learnable parameters, compared to SwinUNETR's 18.4 million. To promote reuse and ensure reproducibility, pre‐trained nnUNet‐R‐LR and SwinUNETR‐R‐LR models will be made openly accessible upon request, along with other tools developed in this study.

Despite the promising findings of this study, several limitations must be addressed. First, both AI and heuristic detection frameworks show high accuracy and specificity, indicating strong performance in classifying non‐artifacts correctly, but the notably lower sensitivity of the deep learning models suggests they may be overly conservative, potentially missing true phase‐binning artifacts. One contributing factor is the severe class imbalance between artifact and non‐artifact voxels, which poses a persistent challenge for model training. This issue could be partially mitigated by increasing the synthetic training set size and adjusting the threshold applied to the probability maps when generating the final predicted artifact masks. Another factor is two‐fold: the presence of interpolation artifacts near phase‐binning artifacts in the training data and the imbalance of phase‐binning versus interpolation artifacts. Each training set of *S1*–*S3* includes approximately 30% of phase images with interpolation artifacts, providing the models with examples of how these artifacts appear so they can learn to avoid them. This mixing of artifact types may be causing the models to adopt more conservative predictions, as interpolation artifacts near the diaphragm can resemble distortions like those caused by phase‐binning artifacts. However, simply increasing the number of interpolation artifact cases to reduce the class imbalance may not resolve this issue and could exacerbate model confusion. Instead, curating clearer distinctions between artifact types in the training data or applying class‐balancing strategies may help mitigate the low sensitivity. The pre‐processing steps applied in previous studies using synthetic interpolation artifacts are often unspecified or unclear.

The previous point leads to another concern on model evaluations and performance comparisons across various studies. While our results demonstrate strong performance of the proposed artifact detection models, we acknowledge important caveats in making direct comparisons to prior methods. The comparisons are not fully standardized, as many existing approaches were tested on different datasets, synthetic generators, scanners, and acquisition protocols, all while reporting inconsistent evaluation metrics. We attempted to mitigate this by including images from six international institutions using various protocols on a diverse set of patients. Additionally, detection methods are developed with specific objectives in mind, like improving user accessibility, performing full‐slice inpainting to restore artifact‐corrupted regions, or evaluating phase‐sorting algorithms. These differing goals necessitate varying levels of sensitivity, specificity, or interpretability, such that performance must be interpreted in the context of each method's intended use case. A method that under‐performs in one metric may still be highly effective for its targeted application.

Another important distinction lies in the type of artifacts used: we evaluate both synthetic and naturally occurring artifacts to assess DL models and optimize the rule‐based method. While synthetic artifacts offer controlled, interpretable benchmarks, they may not fully reflect the complexity of clinical cases. Our models perform best on synthetic data, with real artifact detection remaining more challenging. To address this, we introduced the overfitting parameter ϕ and artifact‐omics comparisons, but further progress will require manually labeled masks that precisely identify voxels affected by phase‐binning and interpolation artifacts.

Our approach complements the diverse landscape of detection models by focusing on interpretability and a dual framework that supports both deep learning and rule‐based detection, but it should be seen as part of a broader toolkit rather than a universal solution. We hope future work will move toward establishing shared benchmarks across diverse artifact types and data sources to enable more equitable comparisons.

## 5 CONCLUSION

We present an artifact generator that produces synthetic data to support both the training of deep learning models and the configuration of a rule‐based method for detecting common 4DCT imaging artifacts. Modified nnUNet and SwinUNETR frameworks are employed to segment phase‐binning artifacts at the voxel level, while the interpretable rule‐based approach simulates interpolation over short slice intervals and identifies low‐difference regions as likely interpolation artifacts based on optimized thresholds. While the artifact generator enables diverse training scenarios, it is the artifact detection models that demonstrate the ability to robustly identify, localize, and ultimately drive improvements in the management of 4DCT artifacts. By deriving artifact‐omics features and introducing a simple localized artifact correction method that significantly enhances artifact‐affected lung masks, we show that certain DL models are not only robust to lung geometries, artifact types, and cancer progressions, but also generalize to true artifacts. Recent artifact correction methods focus on artifact‐affected slices, so by identifying the voxels that play a significant role in the slice classification decision, we contribute to the interpretability of AI in medical applications. In particular, the accurate nnUNet‐R‐LR and SwinUNETR‐R‐LR models have the potential to pave the way for more targeted and exciting artifact correction methods.

## CONFLICT OF INTEREST STATEMENT

The authors have no relevant conflicts of interest to disclose.

## Supporting information

Supporting Information
