# Supplementary material for: Detection of phase‐binning and interpolation artifacts in 4‐dimensional computed tomography imaging using deep learning and rule‐based approaches
Source: Med Phys. 2025 Dec 13;52(12):e70191. doi: 10.1002/mp.70191 (PMC12701711; doi:10.1002/mp.70191)
Supplement: Supplementary file 1 — Supporting Information [file MP-52-0-s001.pdf]

Detection of phase-binning and interpolation artifacts in 4-dimensional computed tomography imaging using deep learning and rule-based approaches

Supplementary File

Jorge Cisneros[1,2*], Nathan H. Feldt[1], Yevgeniy Vinogradskiy[3], Richard Castillo[4], Edward Castillo[1]

[1]Department of Biomedical Engineering, University of Texas at Austin, Austin, TX 78712, USA
[2]Department of Internal Medicine, University of Texas Southwestern Medical Center, Dallas, TX 75390, USA
[3]Department of Radiation Oncology, Thomas Jefferson University, Philadelphia, PA 19107, USA
[4]Department of Radiation Oncology, Emory University School of Medicine, Atlanta, GA 30307, USA

I. Clinical datasets

See Table S-1 for clinical datasets.

*Corresponding author: jorge.cisnerospaz@utsouthwestern.edu
The University of Texas Southwestern Medical Center
5323 Harry Hines Blvd., MC 8830
Building E, Room E5.325
Dallas, TX 75390-8511

Table S-1: An overview of the 9 datasets (first column) used to train, test, and validate our DL models. All phase images are automatically cropped down to the field of view of the lungs (fifth column). Some patients were imaged several times (sixth column), but only the first and last sets of 4DCTs were collected. Each 4DCT set of all but two clinical datasets consists of 10 breathing phases, with only a handful of sets missing one or two phases. The 4DCT sets of *Internal 1* and *4* contain only T00 and T50 breathing phases.

**Datasets**	**4DCT sets**	**Resolution (mm^3)**	**Orig. dimensions**	**Cropped dimensions**	**Pre/post**	**Institution**
4D-Lung	33	0.98 × 0.98 × 3.00	512 × 512 × [77 – 149]	[238 – 306] × [264 – 358] × [77 – 121]	Yes	Virginia Commonwealth University (USA)
CT-PET-VI	12	0.98 × 0.98 × 3.00	512 × 512 × [81 – 99]	[224 – 310] × [288 – 345] × [81 – 99]	No	Royal North Shore Hospital (Australia)
DIR-Lab	10	[0.98 – 1.16] × [0.98 – 1.16] × 2.50	[256, 512] × [256, 512] × [94 – 136]	[232 – 300] × [256 – 351] × [85 – 124]	No	The University of Texas MD Anderson Cancer Center (USA)
POPI	6	0.98 × 0.98 × 2.00	512 × 512 × [139 – 187]	[213 – 322] × [225 – 378] × [121 – 158]	No	Léon Bérard Cancer Center (France)
Internal 1	20	[0.98 – 1.30] × [0.98 – 1.30] × 3.00	512 × 512 × [85 – 144]	[196 – 294] × [236 – 344] × [81 – 117]	Yes	Beaumont Health, University of Colorado (USA)
Internal 2	105	[0.98 – 1.30] × [0.98 – 1.30] × 3.00	512 × 512 × [77 – 160]	[196 – 309] × [233 – 338] × [77 – 114]	Yes	Beaumont Health, University of Colorado (USA)
Internal 3	22	[1.07, 1.17] × [1.07, 1.17] × 3.00	512 × 512 × [121 – 185]	[218 – 283] × [231 – 317] × [102 – 137]	No	The University of Texas MD Anderson Cancer Center (USA)
Internal 4	129	0.98 × 0.98 × 2.00	512 × 512 × [81 – 146]	[217 – 440] × [265 – 488] × [81 – 134]	No	Beaumont Health (USA)
Internal 5	90	[0.58 – 1.27] × [0.58 – 1.27] × [2.50, 3.00]	512 × 512 × [80 – 130]	[187 – 394] × [219 – 490] × [77 – 128]	Yes	University of Colorado (USA)

II. Synthetic generator & artifact masks

The pipeline to insert artifacts begins by producing a binary mask M_g that indicates the stack(s) to replace. However, instead of extracting and directly inserting the slices from $\tilde{I}$ into I, we create a feathered version $\hat{M}_g$ from M_g to allow diffused and blurred artifacts. In particular, we first assign a weight from a Gaussian distribution to each selected slice, decreasing for slices closer to the stack boundaries. The mask is then further smoothed using a Gaussian filter with a chosen standard deviation σ_g for the Gaussian kernel, with typical values between 0.1 and 1.5, determined experimentally. Artifacts in $\hat{I}$ will be more pronounced for $\sigma_g < 1$ and more faded for $\sigma_g > 1$. Relative to the artifact score maximum peak, $R_S \to \infty$ as $\sigma_g \to 0$ and $R_S \to 0$ as $\sigma_g \to \infty$. For this reason, we refer to σ_g as the "artifact visibility" parameter. The non-T00 image $\tilde{I}$ is also modified into $\tilde{I}_g$ with a shift intensity parameter s_i, which slightly shifts axial slices in the coronal and sagittal directions to mimic reconstruction error and noise, especially near the diaphragm.

III. Pre-processing

For supervised training, we require the paired artifact image $\hat{I}$ and its ground-truth artifact mask M_a. Additionally, we introduce the binary region-based mask M_R derived from the lung mask $\hat{M}_\ell$ in order for the DL models to focus on the most relevant regions and ease the computation burden of 3D segmentation. The mask covers the lower half of the lungs where motion-based artifacts are common, while ignoring irrelevant structures as much as possible. It is essentially computed by first zeroing out the top half of $\hat{M}_\ell$, then sequentially performing binary dilation on each axial slice with increasing dilation iterations for each successive slice, starting from the half-way point of the lungs down to the base. This ensures the lung volume and diaphragm regions are accounted for, while disregarding the majority of the heart and several segments of the spine and ribs. Figure S-1 shows the axial, coronal, and sagittal views of a synthetic image with corresponding artifact and region-based masks. Moreover, we perform left and right lung cropping to double the overall dataset size and further focus the models' efforts. All of these steps are optional and can be skipped to analyze their performance impact in an ablation study. Adding to the pre-processing techniques, we lastly apply Z-score normalization to standardize the image intensities across the training set.

IV. True versus synthetic cases

Figure S-2 presents 2 cases with true artifacts and 2 cases with synthetic artifacts from *S3*.

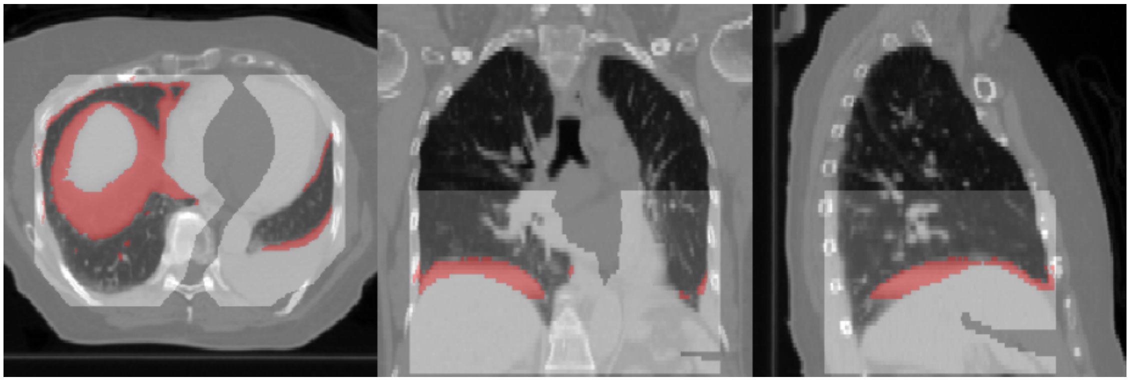

Figure S-1: The paired synthetic case for training: the artifact-affected image $\hat{I}$ and the artifact mask M_a (in red). The region-based masks M_R (as the brighter regions) are also shown.

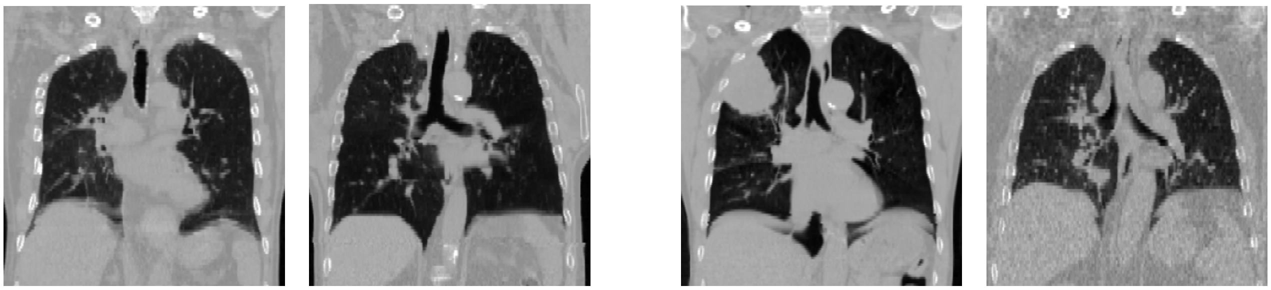

(a) (b) (c) (d)

Figure S-2: Four typical examples of artifact images are grouped, where (a) and (b) have true artifacts and (c) and (d) have synthetic artifacts found in the synthetic dataset *S3*.

V. Clean scans

See Table S-2 for false positive rates on clean scans.

Table S-2: Average false positive rates of the best-performing nnUNet and SwinUNETR detection models on scans *without* artifacts.

**Synth. dataset**	**DL configuration**	**Stack detection FPR**	**Slice detection FPR**	**Voxel detection FPR**
S1	nnUNet-R-LR	0.0047	0.0047	0.0001
	SwinUNETR	0.0406	0.0406	0.0002
S2	nnUNet-R-LR	0.0177	0.0177	0.0002
	SwinUNETR-R-LR	0.0418	0.0418	0.0002
S3	nnUNet-R	0.0089	0.0085	0.0001
	SwinUNETR-R	0.0458	0.0458	0.0002
